# Delta/Theta band EEG activity shapes the rhythmic perceptual sampling of auditory scenes

**DOI:** 10.1038/s41598-021-82008-7

**Published:** 2021-01-27

**Authors:** Cora Kubetschek, Christoph Kayser

**Affiliations:** 1 Biological Psychology and Neuropsychology, University of Hamburg, Hamburg, Germany; 2 Department for Cognitive Neuroscience, University of Bielefeld, Bielefeld, Germany

**Keywords:** Neuroscience, Psychology

## Abstract

Many studies speak in favor of a rhythmic mode of listening, by which the encoding of acoustic information is structured by rhythmic neural processes at the time scale of about 1 to 4 Hz. Indeed, psychophysical data suggest that humans sample acoustic information in extended soundscapes not uniformly, but weigh the evidence at different moments for their perceptual decision at the time scale of about 2 Hz. We here test the critical prediction that such rhythmic perceptual sampling is directly related to the state of ongoing brain activity prior to the stimulus. Human participants judged the direction of frequency sweeps in 1.2 s long soundscapes while their EEG was recorded. We computed the perceptual weights attributed to different epochs within these soundscapes contingent on the phase or power of pre-stimulus EEG activity. This revealed a direct link between 4 Hz EEG phase and power prior to the stimulus and the phase of the rhythmic component of these perceptual weights. Hence, the temporal pattern by which the acoustic information is sampled over time for behavior is directly related to pre-stimulus brain activity in the delta/theta band. These results close a gap in the mechanistic picture linking ongoing delta band activity with their role in shaping the segmentation and perceptual influence of subsequent acoustic information.

## Introduction

Perception and cognition are controlled by rhythmic activity in the brain^1–3^. These rhythmic processes can reflect directly in behavioral data, such as periodic changes in reaction times or measures of perceptual accuracy relative to stimulus onset^4–7^. More frequently, they are revealed by systematic relations between signatures of rhythmic brain activity and measures of performance, such as changes in accuracy or sensitivity with the power or timing of pre-stimulus activity^8–10^. Concerning hearing, several studies have shown that performance varies with pre-stimulus activity below 10 Hz. For example, participants’ ability to detect brief acoustic targets or to discriminate two subsequent tones varied with the power and phase of brain activity below about 4 Hz^8,9,11–14^. The apparent match between the time scales of perceptual sensitivity and those at which neural activity shapes hearing^15,^^16^ is seen as strong support of a rhythmic mode of hearing. Such a rhythmic mode could facilitate the amplification of specific (e.g. expected) stimuli and mediate the alignment of endogenous neural activity to the regularities of structured sounds such as speech^17–19^.

A critical prediction based on these studies, and motivated by a link between rhythmic network activity and the functional gain of individual neurons, is that perception should sample acoustic information rhythmically rather than continuously over time^2,^^10,^^17,^^20,^^21^. Thereby, also information in longer soundscapes that are devoid of an explicit temporal structure should be weighted at precisely those timescales at which rhythmic brain activity is predictive of behavior (i.e. between the delta and theta bands between about 1 and 4 Hz). Studies on speech have provided evidence in favor of this hypothesis^18,^^22–25^, e.g. by showing that delta band activity serves the chunking or segmentation of speech on a sentence-level time scale^15,^^18,^^26^ while theta band activity reflects the processing of syllable-scale information. However, the underlying processes may possibly be specific to speech, which is intrinsically predictive on multiple time scales. Other studies have used periodic sounds to entrain rhythmic neural processes and have shown the persistent and periodic influence of these on behavior for several cycles even after the offset of the entraining sound^27,^^28^. However, this does not demonstrate a direct influence of pre-stimulus and possibly spontaneous brain activity on a subsequent rhythmic mode of listening.

To more broadly address the question of whether listening samples acoustic information based on rhythmic processes in the delta or theta time scales, we have previously designed a paradigm allowing the quantification of the moment-by-moment influence of acoustic evidence on perceptual judgments^29^. In that earlier study, we found evidence in favor of a rhythmic listening mode in human participants. However, by design that study did not link the rhythmic weighting of acoustic evidence to brain activity and made the strong assumption that the temporal weighting profile is idiosyncratic across trials^29^. That is, it assumed that the relative perceptual sampling phase is consistent on a trial by trial basis. However, if the excitability of auditory pathways is controlled by (rhythmic) pre-stimulus brain activity^30,^^31^, this assumption could be violated: the temporal perceptual weighting profile at which momentary acoustic evidence is sampled should change on a trial by trial basis relative to the trial-wise pattern of pre-stimulus brain activity.

Here we directly tested this prediction by asking whether the rhythmic behavioral use of acoustic information is directly related to pre-stimulus activity. To probe this, we combined psychophysical reverse correlation with EEG recordings obtained while human participants judged the direction of frequency sweeps in pseudo-random soundscapes of 1.2 s duration. We first reproduced our previous results providing evidence for a rhythmic perceptual sampling of extended soundscapes at a frequency of about 2 Hz. Then, we show that the relative timing of these perceptual weights is significantly related to the power and phase of pre-stimulus EEG activity at a similar time scale, with the perceptual weights of opposing phase bins differing by about 90 degrees.

## Methods

### Participants

The experiment combined a previously described behavioral task with electroencephalography (EEG) recordings in 20 participants (11 females; 19–32 years old). The study was conducted in accordance with the Declaration of Helsinki and was approved by the ethics committee of Bielefeld University. Data was collected with participants’ written informed consent. Participants reported normal hearing and received monetary compensation of 10 Euro/hour. During the experiment they sat in an electrically and acoustically shielded room (Ebox, Desone, Germany).

### Stimuli

**Figure 1:**
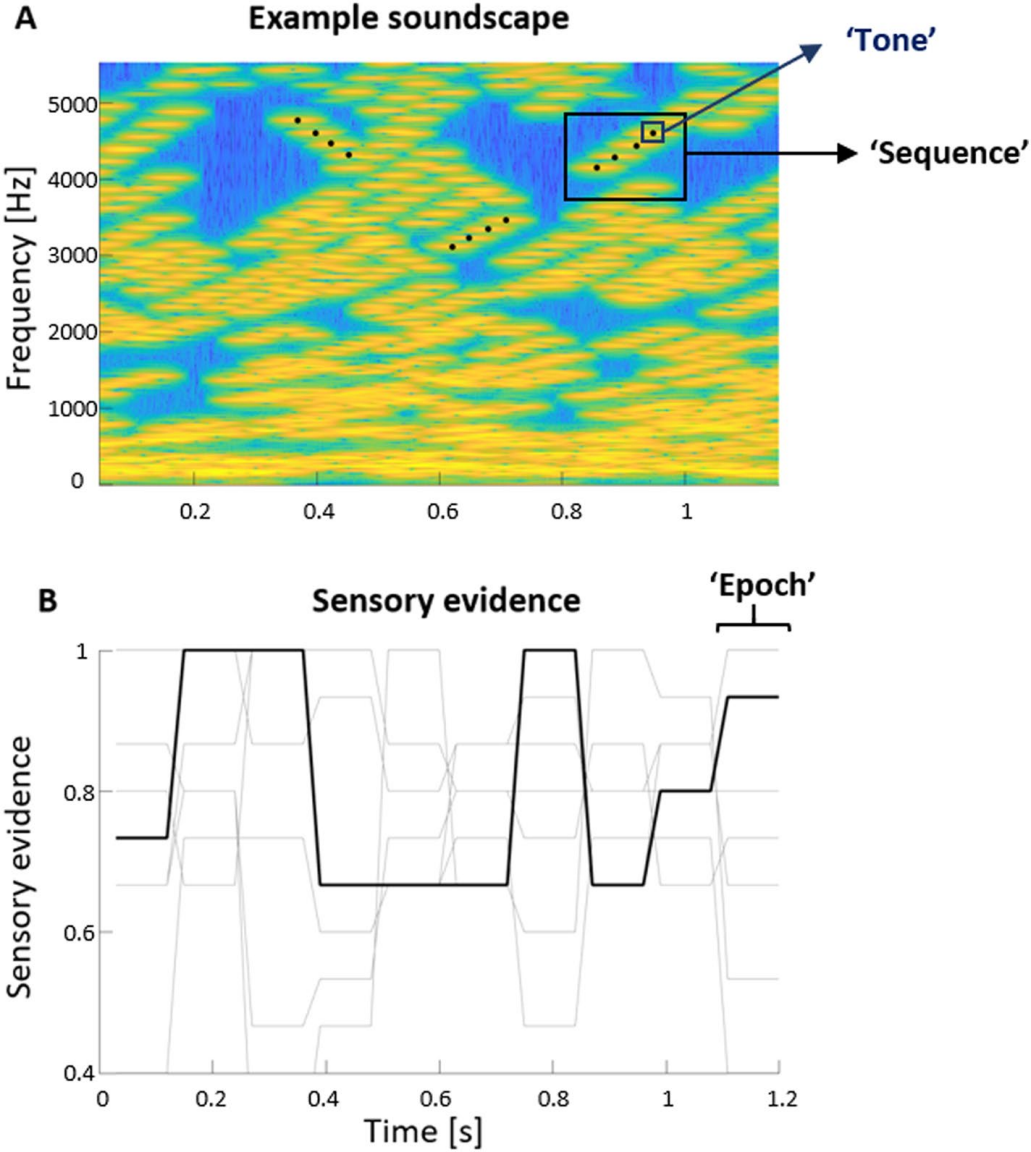
Example soundscape. (**A**) Time–frequency representation of one soundscape with an overall increasing frequency sweep. Black dots indicate the four consecutive tones of three selected sequences. Yellow colors indicate higher sound levels. (**B**) The 'sensory evidence' for the soundscape in A (black line) and other example soundscapes (grey lines). An evidence value of 0.5 indicates ambiguous evidence, a value of 1 that a fully coherent soundscape in which all tone sequences increase.

The stimuli and task have been described in detail before^29^. The stimuli were presented via headphones (Sennheiser HD200 Pro) at an average intensity of 65 dB SPL. Each stimulus was composed of 30 simultaneously presented sequences of four tones each, whereby each sequence either increased or decreased in frequency over the four tones (Fig. 1A). Each tone had a duration of 30 ms. The starting frequency of each sequence was drawn independently between 128 and 16,384 Hz and increased or decreased in steps of 20 cents. The starting position within each sequence (1 to 4) of the initial 30 sequences were selected at random to ensure that the frequencies and the start/end times of each sequence were independent across the 30 sequences. Also, the exact starting times of individual tones within a sequence varied up to 30 ms. To create the impression of an overall frequency-sweep over time, the proportion, termed ‘coherence’, of in/decreasing tone sequences was systematically varied. This coherence could vary between 0 (indicating that half the tone sequences increased, while the other half decreased) and 1 (indicating that all swept in the same direction). Each trial in the experiment, and hence each soundscape, was characterized by the direction of change (increasing or decreasing) and the associated coherence. This design allowed us to vary the amount of sensory evidence about the direction of sweep between and within a trial around each participant’s threshold (see below). Specifically, each soundscape of 1200 ms duration was divided into ten ‘epochs’, each lasting 120 ms (see Fig. 1B). The coherence for each epoch was drawn randomly and independently from a Gaussian distribution centered around the participants’ threshold and with a standard deviation of 0.2. To obtain the stimulus for a given trial we first determined the sweep direction (increasing or decreasing) and then sampled the coherence for each epoch and subsequently generated the sequences of pure tones to fit those parameters.

We quantified the temporal modulation spectrum of these soundscapes as done previously^29^. First, we computed the band-limited Hilbert envelope of each soundscape in 10 logarithmically spaced bands between 100 and 12 kHz. Then we derived the average temporal modulation spectrum for each band across soundscapes and participants.

### Task and experimental design

The participant’s task was to report the perceived direction of frequency change (‘sweep’) of the stimulus after each trial as accurately as possible. Each experiment consisted of five blocks with 200 trials each, resulting in 1000 trials per participant. The inter-trial intervals had a duration of 1100–1600 ms (uniform random distribution). Each trial started with a fixation cross after which (800–1100 ms uniform distribution) the soundscape started. Participants could take breaks in between blocks. This design corresponds to Experiment 3 in Kayser et al. 2019^29^, except that here we obtained 1000 trials (rather than 800).

Before the actual experiment, we determined participants’ perceptual thresholds using three interleaved 2-down 1-up staircases that each varied the coherence of the presented soundscapes (starting at different initial coherence values of 0.15, 0.4 and 0.8 respectively, with initial step sizes of 0.1). An average of six reversals (excluding the initial four) was calculated from each staircase, and the resulting three coherence thresholds were averaged to yield the final participant’s threshold for judging the direction of frequency sweeps in these soundscapes. Across participants the obtained thresholds were 0.32 ± 0.05 (mean ± s.e.m.).

The behavioral performance was quantified as the fraction of correct responses and using two measures from signal detection theory, sensitivity (d’) and bias (c)^32^, by dividing the trials according to sweep direction.

### Analysis of psychophysical weights

The soundscapes were designed to allow the application of psychophysical reverse correlation to quantify the influence of the momentary sensory evidence (deviation from an ambiguous sweep direction) on participant’s responses^33,^^34^. For this analysis, the sensory evidence was operationally defined as the signed difference between the half the actual coherence value and a value of 0.5: an evidence of 0 defined a perfectly coherent decreasing soundscape, a value of 1 a perfectly coherent increasing soundscape, and a value of 0.5 an ambiguous soundscape (c.f. Fig. 1).

For each participant we derived a perceptual weighting profile as follows: we split trials according to the participants’ response and sweep direction. For each response we calculated the average motion evidence and converted their difference into a within-participant z-score based on a distribution of 4000 weights obtained by randomizing the alignment of stimuli and responses^35,^^36^. A weight of zero indicates no influence of the stimulus in that epoch on participant’s responses, while positive values indicate a positive relation between sensory evidence (i.e. the amount of sweep coherence and the direction of sweep) and the participant’s response. Note that this calculation assumes that the time course of the perceptual weights is consistent across trials within a participant, as the reverse correlation assigns a fixed weight to each epoch. To relieve this assumption, the main analysis in this study derived the perceptual weights for subsets of trials that were chosen based on the amplitude or phase of EEG signals in a pre-stimulus period, as described below.

To probe whether these perceptual weights exhibited a systematic temporal structure, we proceeded as previously^29^. We first extracted non-rhythmic structures such as an offset, a linear ramp and u/v-shaped time courses fixed to the stimulus duration. The u/v shaped component was modeled as cos(2 ∗ pi ∗ t ∗ fexp), with fexp = 1/stimulus duration, and reflects a potential (de-) emphasis of the middle proportion of the stimulus. These three components were termed ‘trivial’, as they do not relate to the specific hypothesis of genuine rhythmic structure at relevant timescales above 1 Hz. We then quantified whether a rhythmic component at a frequency above 1 Hz significantly contributes above these trivial components to the time course of the perceptual weights. For this we compared regression models featuring only the trivial components with models additionally including a rhythmic component, defined by sine and cosine components of a variable frequency between 1.1 and 4 Hz. We tested this specific frequency range, as frequencies above or below were outside the temporal sampling range defined by the duration of these soundscapes and the temporal resolution at which perceptual weights were calculated (120 ms). This temporal resolution is defined by the duration of the epochs between which the motion coherence was randomized within a trial (see above). To compare regression models we followed two slightly distinct approaches^29,^^37^. First, for each model (with and without rhythmic component) we derived its log-evidence obtained from the regression for individual participants. Model comparison was then based on the group-level log-evidence (assuming that participants contribute independently)^37–39^. We additionally computed the exceedance probability of either the trivial model or the trivial plus rhythmic model to better explain the data using a bootstrapping procedure, and we computed model frequencies, which indicate the proportion of participants for which either model explains the data best^40^. In a separate analysis we used a Monte-Carlo approach for model fitting and compared models based on the Watanabe-Akaike information criterion (WAIC), which also captures the out-of-sample predictive power when penalizing each model^38^. This calculation was implemented using the Bayesian regression package for Matlab^41^, using 10,000 samples, 10,000 burn-in samples and a thinning factor of 5. We did not expect clear differences between these two approaches for model comparison, but each offers a different tradeoff of capturing in- and out-of-sample predictive power^38^.

### EEG recordings and analysis

EEG was recorded continuously using a 64-channel ActiveTwo system (Biosemi), with reference electrodes located occipital-parietal at a frequency of 1024 Hz. Electrodes to record the electro-oculograms (EOG) were put below and next to the lateral canthus of both eyes.

The EEG data were analyzed using Matlab (R2017a; TheMathWorks) and the fieldtrip toolbox, version 20190905^42^. The raw data was filtered (between 0.6 and 70 Hz; 3rd-order Butterworth filter) and re-sampled to 150 Hz. Trials were rejected if the amplitude in central electrodes exceeded ± 175 µV. An average of 21.1 ± 8 (SEM) trials per participant were rejected. Few bad channels were interpolated based on all neighboring channels^43^. Furthermore, artefacts were identified and rejected, using the data from the EOG channels, and based on an independent component analysis (ICA). Artifacts were identified as in our previous studies^44,^^45^ following definitions provided in the literature^46,^^47^ and included poor electrode contacts, frontal artifacts induced by blinks or eye movements, and temporal muscular artifacts. On average we removed 17 ± 1 (mean ± s.e.m.) components. Our main analysis focused on the relation between rhythmic brain activity prior to the stimulus and the perceptual weights. To quantify this, we first performed a time–frequency analysis on single trial EEG activity in a time window prior to stimulus onset (− 1 to 0 s). To avoid contamination by post-stimulus activity, we time-mirrored the epoched data and applied a Hanning window to fade out the stimulus period^48^. Time–frequency resolved activity was obtained using Morlet wavelets (4 cycles width) between 2 and 13 Hz, from which we derived the time-varying power and phase of each frequency band. This range was chosen based on the relevant time scales revealed by previous work^8,^^9,^^24,^^49–54^ (or for review see^55^) and the available pre-stimulus data epoch.

### Linking EEG and behavior

To link pre-stimulus EEG activity and behavior, we first quantified the relation between measures of perceptual performance and EEG power and phase in the pre-stimulus period. For this, we divided trials into those with high or low pre-stimulus power (based on a median split) for each participant, electrode, frequency and pre-stimulus time point. Then we quantified behavioral performance separately for trials with low or high power. To test for a statistical effect, we computed a two-sided t-test across participants between trials with low- and high power for each channel, time point and frequency. Because this involved a large number of dimensions, we reduced this dimensionality as follows: we first used the analysis focusing on the fraction of correct responses to define a suitable time point to extract power by determining that time point containing the most significant (at an uncorrected p < 0.05) number of channels across frequencies. We then extracted the averaged power in a time window around this time point (− 200 ± 100 ms). Subsequently we tested for a significant relation between power and behavioral sensitivity or bias using cluster-based permutation statistics (see below). To visualize the dependency of behavior on power we also divided the trials according to pre-stimulus power into four bins, with each bin containing the same number of trials (c.f. Fig. 3D).

We used a similar two-stage procedure to test for a relation between EEG phase and behavior. First, we split trials into correct or incorrect responses and contrasted these using the phase opposition sum (POS)^56^. The POS was computed for each participant individually and these were combined across participants using the Stouffer Method using the PhaseOpposition toolbox^56^. To reduce dimensionality, we calculated the number of significant channels (at p < 0.05, uncorrected) for each time point and frequency and determined the time with the highest number of significant channels across frequencies. This time point (− 320 ms) was then used for the subsequent analysis of EEG phase. For the main analysis we then grouped trials according to the phase at this time point, similar to the analysis of power. However, because the division of phase in two bins is arbitrary, we repeated this analysis using four different division boundaries to split the circular phase range in two groups (dividing trials along boundaries at 0 and π, along boundaries at π/4 and − 3 π/4, etc.). Then we computed the absolute difference in sensitivity or bias between opposing phase bins, selected for each participant, electrode and frequency the phase division yielding the strongest effect, and used cluster-based permutation statistics to determine significant effects.

### Linking EEG and perceptual weights

**Figure 2:**
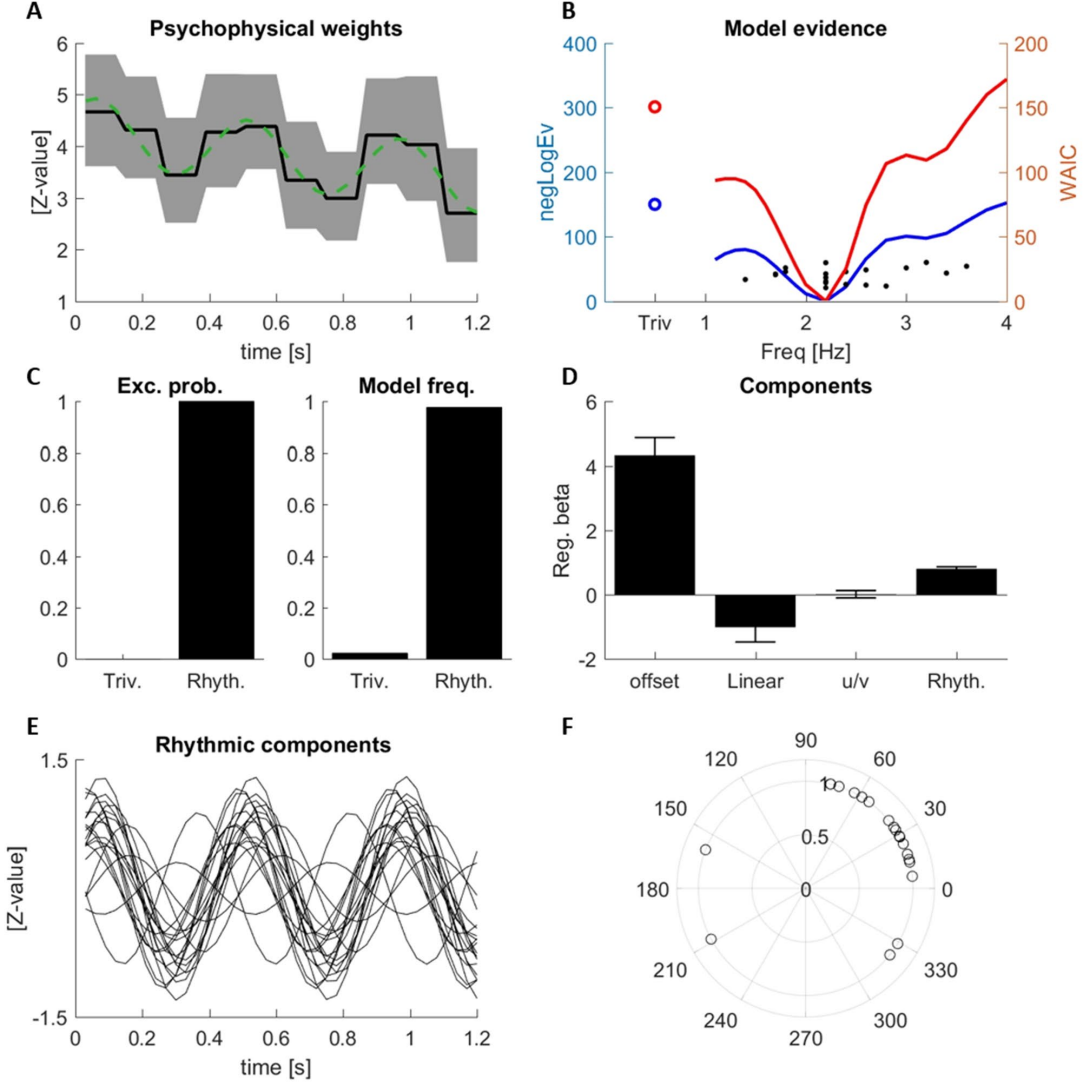
Perceptual weights. (**A**) Group-averaged perceptual weights (black line), two-sided 95% bootstrap confidence interval (grey area) and the best fitting models (group-average; green). (**B**) Model comparison between the trivial model (circle) and rhythmic models at different frequencies (lines) based on the group-level negative log-evidence (blue) and the WAIC (red). Dots indicate participant’s individual best frequencies (from negLogEv). (**C**) Exceedance probabilities and model frequencies comparing the trivial and a rhythmic model at the best rhythmic group-level frequency (2.2 Hz). (**D**) Regression betas for each model component (mean, s.e.m.): the overall offset, the linear slope, a u- or v-shaped contribution over the full duration and the rhythmic component; See Methods for details. For the rhythmic component the root-mean-squared amplitude of the combined sine and cosine components at 2.2 Hz is shown. (**E**) Rhythmic components of the best model for each individual participant. (**F**) Phase of the rhythmic component for each participant.

To test whether and how pre-stimulus power and phase affect the sampling of acoustic information, we quantified the relation between these and the perceptual weights. To do so, we recomputed these weights and their trivial and rhythmic components obtained using regression separately for trials falling into either of the two bins defined based on EEG power or phase. This was done for each participant, frequency band and electrode separately, with phase and power extracted at the respective optimal time points described above. We then focused on the different trivial and rhythmic components of these weights defined above, considering the rhythmic component at the best group-level frequency of 2.2 Hz (as revealed in Fig. 2). We asked whether these components differed in amplitude (or for the rhythmic component, additionally differed in phase) between trials characterized by the two bins of EEG power or phase.

### Statistics

Statistical tests for EEG data were based on a two-level procedure and used cluster-based permutation procedures to correct for multiple comparisons across electrodes and frequency bands, and for phase, additionally for the inclusion of four potential divisions of phase into two bins. To control for multiple comparisons across performance indices (e.g. d’ and bias; or different components of the perceptual weights) we used the Benjamini & Hochberg false discovery rate^57,^^58^ to threshold significant clusters at a corrected p-value of 0.01.

To test for a significant relation between EEG power and behavior, we first used a paired t-test to contrast sensitivity (or bias) between power bins across participants. Then, we entered the respective t-values (thresholded at a two-sided level of p < 0.05) into a permutation procedure, relying on 2000 permutations of the effect sign across participants, using the max-sum as cluster-forming statistics and considering only clusters exceeding a minimal cluster size of two^59^. The same procedure was used to test the relation between EEG power and parameters derived from the behavioral templates (except the phase of the weighting function; see below).

For EEG phase we used a similar statistical procedure. However, as the split of phase into two bins is arbitrary, we considered for each electrode, frequency and participant four potential divisions of phase into two bins. Because the label of each bin, and hence the sign of the difference of effects between bins is arbitrary, we computed the absolute difference between phase bins of the variable of interest. We then selected, for each electrode, frequency and participant the one (of four) phase divisions with the largest effect and computed the average across participants. We then compared this true group-level average effect to a distribution of group-level effects obtained from a permutation of trial labels and behavioral data and accepted as significant effects exceeding the 95th percentile of the randomized distribution. We then applied cluster-based permutation procedure as above. The effect of EEG power or phase on the phase of the perceptual weights was tested similarly, by using the absolute value of the change in phase of the weighting function between the two bins derived from EEG power or phase.

## Results

### Behavioral results

Participants were judging the perceived direction of frequency sweep in 1.2 s long soundscapes. These soundscapes (Fig. 1A) consisted of 30 simultaneous tone sequences, which varied in frequency and the amount of sensory evidence about sweep direction, defined by the coherence of the tone sequences. These soundscapes were designed to allow the quantification of the stimulus–response relation using psychophysical reverse correlation. The resulting perceptual weights are shown in Fig. 2A and reflect the influence of the momentary sensory evidence on behavior. The group-level weights were significant for all time points (at p < 0.05, group-level bootstrap test).

As in our previous study, we investigated the temporal pattern of these perceptual weights by probing their temporal structure using regression modeling. In particular, we asked whether these weights feature rhythmic temporal structure at a time scale of above 1 Hz. To this end, we modeled weights based on three trivial components: a constant offset, a linear slope and a u/v-shaped component time-locked to the soundscape duration. We then added a rhythmic component with a variable time scale between 1.1 and 4 Hz to these and asked whether addition of this rhythmic component significantly improved the descriptive power for these perceptual weights. For each participant, we quantified the contribution of these four components to the participant-specific weights using regression models. We then computed the log model evidence for either a regression model comprising only the three trivial components and a model additionally including the rhythmic component at varying frequencies (Fig. 2B, blue curve). In a separate analysis, we performed the same model comparison using Monte-Carlo simulations used to derive the WAIC criterion (Fig. 2B, red curve). Both analyses consistently revealed that including a rhythmic component at 2.2 Hz provided the highest explanatory power compared to all other frequencies tested. In particular, a group-level model comparison between the trivial model and the rhythmic model at the best group-level frequency (2.2 Hz) revealed that the model including the rhythmic component explained the data significantly better than a model without: the group-level log-evidence was clearly in favor of the rhythmic model (Delta_neglogEv = 147; exceedance probability p_ex_ = 1; model frequency across participants 0.975; Fig. 2C). The same conclusion was supported by a model comparison based on the WAIC (D_WAIC = 148). This result confirms our previous data t obtained in a separate group of participants (Experiment 3 in^29^).

To illustrate these four contributions to the perceptual weights, the green dashed line in Fig. 2A shows the best (group-level) model fit to the actual data and Fig. 2D displays the amplitudes (regression beta’s) for the different components: offset (mean = 4.35; SEM = 0.528), linear decrease (mean = − 1.002; SEM = 0.461), u/v-shaped component (mean = 0.026; SEM = 0.117), and the rhythmic component (mean = 0.804; SEM = 0.059). Figure 2E displays the rhythmic component for each participant individually, illustrating the consistency of the rhythmic perceptual weight for most participants. As shown in Fig. 2F these rhythmic components share a common phase across most participants.

To confirm whether the behavioral data fit the expectations given the experimental design around participant’s thresholds, we used signal detection theory, dividing trials by sweep directions into two classes. Hit rates were around 0.72 as expected (median = 0.716), sensitivity was above 1 for most observers (Median = 1.173; max = 1.81; min = 0.632) and the response criterion revealed no bias (median = -0.01; max = 0.299; min = -0.619).

### Analysis of EEG data

The analysis of EEG data was designed to probe whether the perceptual weights reflecting the influence of acoustic evidence on participants’ behavior were related to the state of brain activity prior to the stimulus. That is, we asked whether (statistically) the shape of these weights differed depending on the state of brain activity prior to the stimulus. Such a dependency could for example reveal whether the overall strength of perceptual sampling (offset), or the strength of the rhythmic contribution differs between trials with particularly high or low power. It could also reveal whether the timing of rhythmic sampling (the weight’s phase) differs between trials preceded by a particular phase in a specific EEG band.

Addressing these questions using statistics required us to first reduce the complexity of this analysis by removing one (least-interesting) dimension: the precise time point prior to the stimulus used to characterize brain activity. We hence implemented a first analysis determining the time points that seemed most promising to capture any dependency between EEG power (or phase) and behavior. To do so for EEG power we computed the difference in the fraction of correct responses between trials with high or low power and quantified how many channels exhibited a significant difference in performance (at p < 0.05) at each frequency and time point. This revealed a cluster of more than 60% significant channels between 300 and 100 ms before stimulus onset, with a peak around 200 ms. We hence used the average over this time window for the subsequent analysis of EEG power. For EEG phase, we calculated the phase opposition sum (POS) as an index of whether pre-stimulus phase differs between trials with correct and wrong responses^56^. Calculating the number of channels with a significant group-level effect revealed a peak 320 ms before stimulus onset, which was used for the subsequent analysis of EEG phase.

### Linking EEG activity and behavior

**Figure 3:**
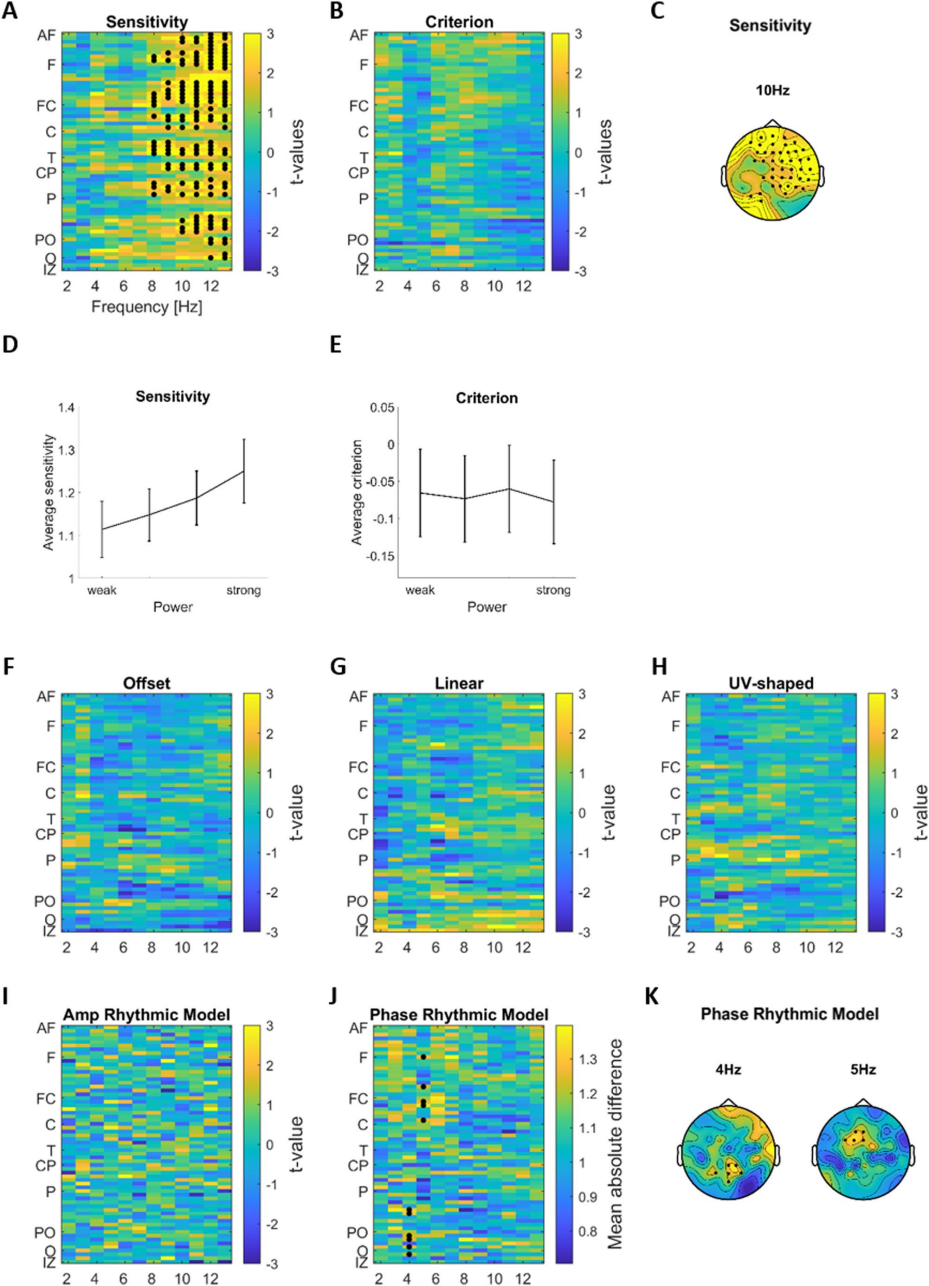
Linking EEG power with behavior and perceptual weights. (**A**,** B**) Difference in perceptual sensitivity and criterion between trials with high or low pre-stimulus power (group-level t-values; paired t-test). (**C**) Topography of the significant electrodes for sensitivity overlaid on the t-map at 10 Hz EEG. (**D**,** E**) Sensitivity and criterion across participants and significant channels (from the cluster for sensitivity in panel A; mean and s.e.m. across participants) as a function of power (four equi-populated bins). (**F–I**) Difference in the prominence (amplitude) of the different components of the perceptual weights between trials with high and low pre-stimulus power (group-level t-values; paired t-test), as a function of EEG frequency and EEG electrode. (**J**,** K**) Difference in relative phase of the rhythmic perceptual component between trials with high and low power (group-level average absolute difference). Electrodes from two significant clusters are marked with black dots (first level significance at p < 0.05, cluster significance at p < 0.01 FDR).

To test for a relation between EEG power and behavior, we contrasted perceptual sensitivity and bias between trials with particularly high or low EEG power (Fig. 3A–E). For sensitivity this revealed a significant positive cluster between 8 and 13 Hz (t_clus_ = 449.016 p = 0.002, 42 channels over fronto-central areas), which was also significant after correcting for all comparisons using the False Discovery Rate (p < 0.01). The localization of this cluster is visualized in Fig. 3C by highlighting all significant electrodes in one topography. For bias we did not find a significant effect (at p < 0.01 uncorrected, Fig. 3B). To visualize the relation between EEG power and behavior in more detail, Fig. 3D, E show sensitivity and bias as a function of power, obtained by dividing trials according to power into four bins.

**Figure 4:**
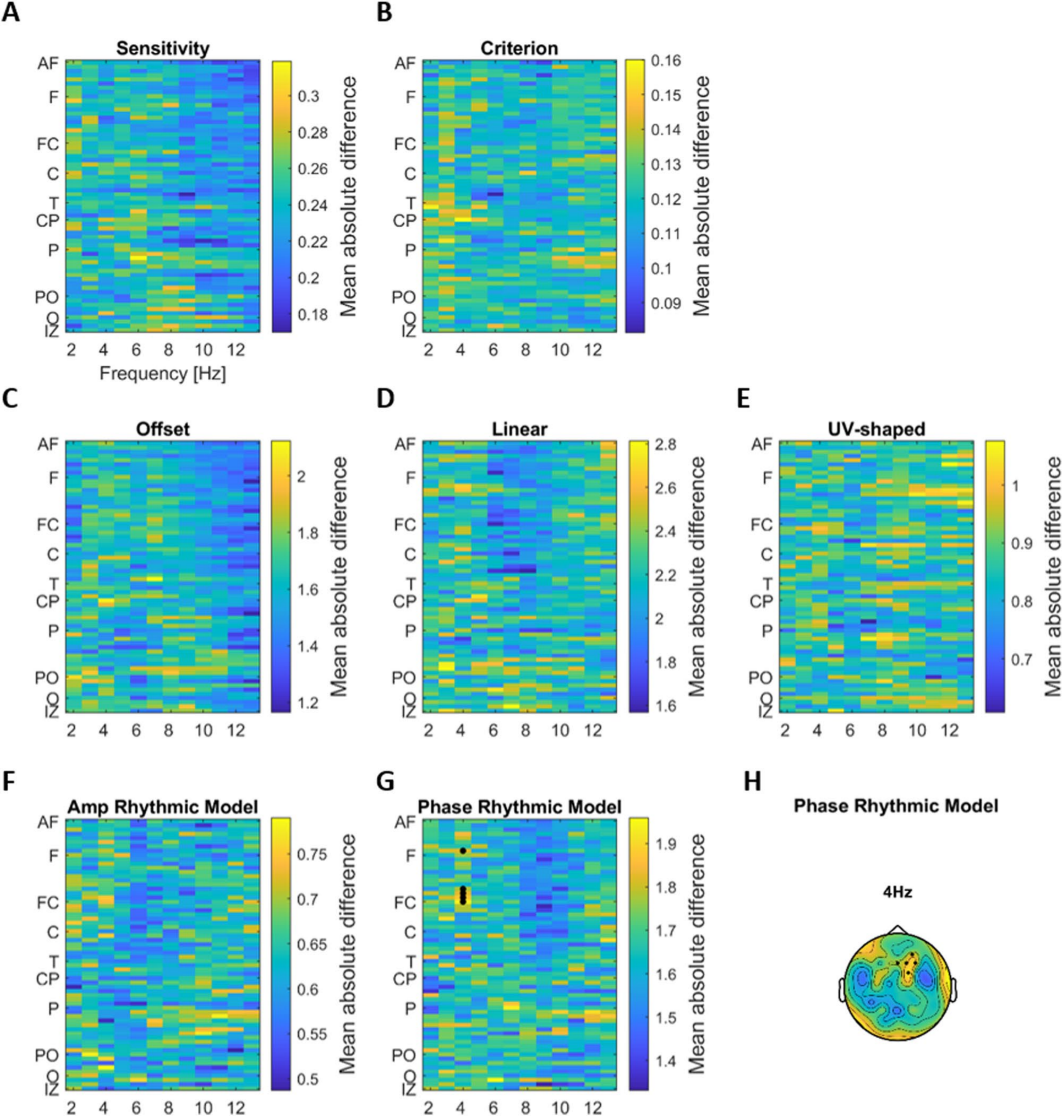
Linking EEG phase with behavior and perceptual weights. (**A**,** B**) Group-level average of participants-wise differences in perceptual sensitivity and criterion between trials with opposing EEG phase. (**C–G**) Group-level average of participants-wise differences in regression betas of each component of the perceptual weights between trials with opposing EEG phase, as a function of EEG frequency and EEG electrode. (**H**) Topographic representation of the group-level average of participants-wise differences in the phase of the rhythmic contribution to perceptual weights. Electrodes from significant clusters are marked with black dots (first level significance at p < 0.05, cluster significance at p < 0.01 FDR).

We performed a similar analysis for EEG phase (Fig. 4A,B). This revealed no significant effects (at p < 0.01 uncorrected).

### Linking EEG activity and perceptual weights

To test whether the perceptual sampling of acoustic information is affected by pre-stimulus brain activity, we asked whether the perceptual weights differ between trials characterized by high or low EEG power, or by different phase states in a particular frequency band. We tested such relations for each of the four model components used to describe the weighting function (offset, linear slope, u/v profile, and the rhythmic component). In doing so, we focused on the amplitude of all components and for the rhythmic component in addition on the relative phase of this. The latter analysis allowed us to directly test whether for example the pre-stimulus phase affects the phase of the rhythmic perceptual sampling. Statistical cluster-based permutation tests were corrected for multiple frequencies and considering multiple division of phase into two bins using the max-statistics and for multiple contrasts using the FDR.

For pre-stimulus EEG power, we found no significant effects for the trivial model parameters and the amplitude of the rhythmic model (at p < 0.01 uncorrected; Fig. 3F–I). However, we found two significant clusters for the phase of the rhythmic model: one at 4 Hz over parieto-occipital electrodes (t_clus_ = 11.787 p = 0.0017, 6 channels, Fig. 3J,K) and one at 5 Hz over fronto-central electrodes (t_clus_ = 10.473 p = 0.005, 5 channels), which were also significant after correcting for all contrasts using the FDR.

For the pre-stimulus EEG phase, these tests revealed no significant effects on the three trivial model parameters (offset, linear and u/v-shaped) or the amplitude of the rhythmic model (at p < 0.01 uncorrected) (Fig. 4C–F). However, a significant cluster emerged for the influence of the 4 Hz EEG phase on the phase of the rhythmic perceptual component over frontal electrodes (Fig. 4G, H; t_clust_ = 11.998, p < 0.001, 5 channels). This indicates that the relative phase by which perception samples acoustic information around 2 Hz changes with the 4 Hz EEG phase over frontal sites. Given that both EEG phase and EEG power at 4 Hz revealed a significant relation to the phase of rhythmic perceptual sampling, we asked whether the strength of both effects was correlated across participants. A non-parametric correlation turned out to be not significant (rank- correlation: r = 0.4331, p = 0.0565, 95th bootstrap-CI [− 0.0144, 0.7097]).

**Figure 5:**
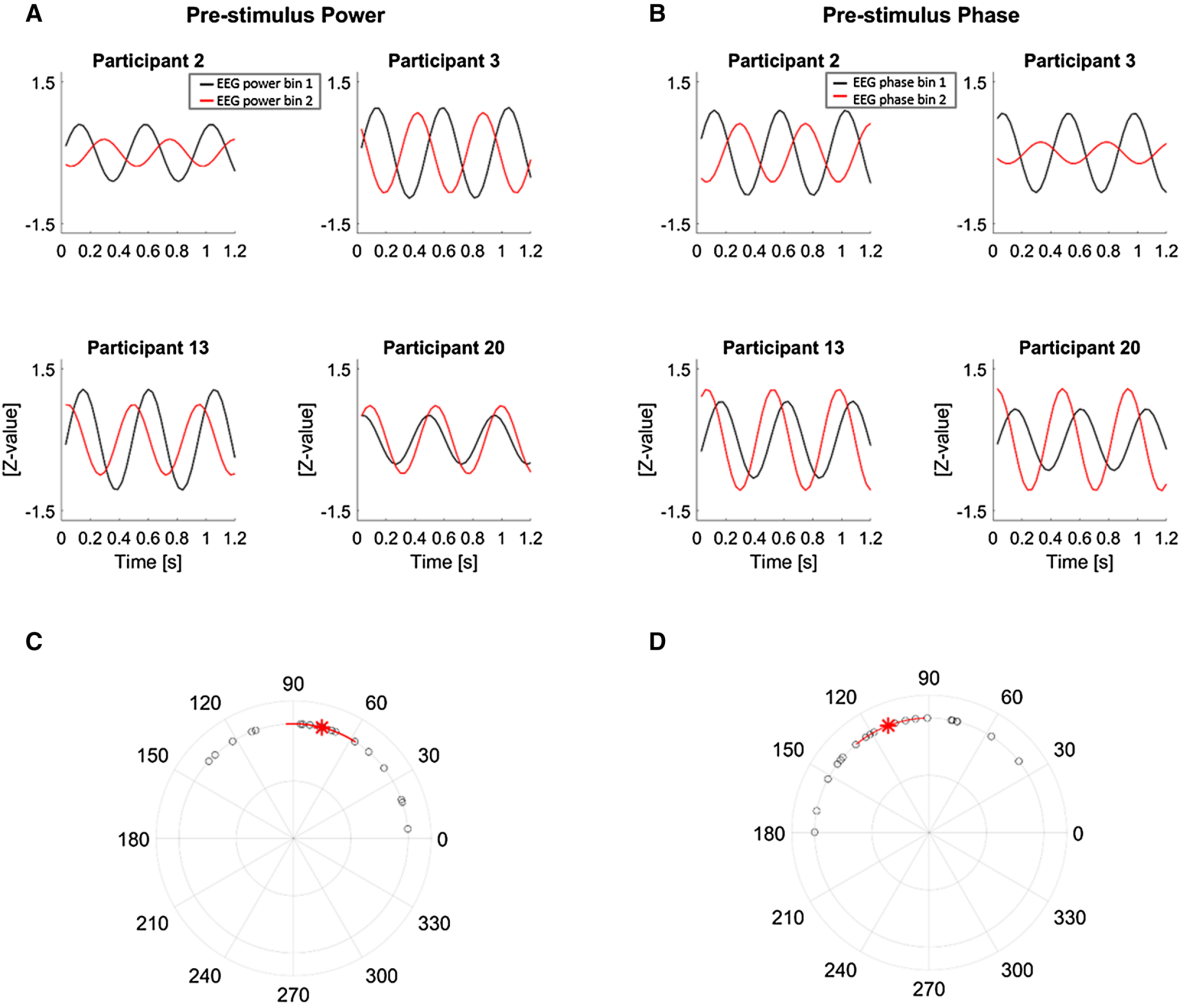
Visualization of the change in rhythmic perceptual sampling with EEG power and phase. (**A**,** B**) Examples of the rhythmic components (at 2.2 Hz perceptual sampling frequency) of the perceptual weights for four participants reconstructed using the respective participant’s specific bins for EEG power (**A**) and phase (**B**). For this analysis we considered the EEG power and phase bins at 4 Hz for each participant yielding the largest difference, derived at EEG electrode Pz for power and FC2 for phase. (**C**,** D**) Absolute phase differences in the rhythmic component of the perceptual weights between the EEG power (**C**) and phase (**D**) bins for each participant. The group-mean is marked with the red star and 95% confidence intervals (CI) are marked by the red lines.

To visualize the relation between pre-stimulus EEG power or phase and the perceptual weights we reconstructed the rhythmic component of the perceptual weights for individual participants using the participant-specific division of trials by EEG power or phase and obtaining the respective regression models on the perceptual weights. The four examples shown in Fig. 5A, B, illustrate the rhythmic component of the perceptual weights. These illustrate both the change in perceptual sampling phase with EEG power and EEG phase, but also reveal the heterogeneity of the effect across participants. On average across participants the absolute phase shift in rhythmic perceptual sampling with EEG power was 75.68° (95th CI of the mean [57.19, 94.15]) (Fig. 5C). The absolute phase shift in rhythmic perceptual sampling across opposing EEG phase bins was 110.98° (95th CI of the mean [92.33, 129.64]) (Fig. 5D).

### Absence of rhythmic structure in EEG and acoustic power

**Figure 6:**
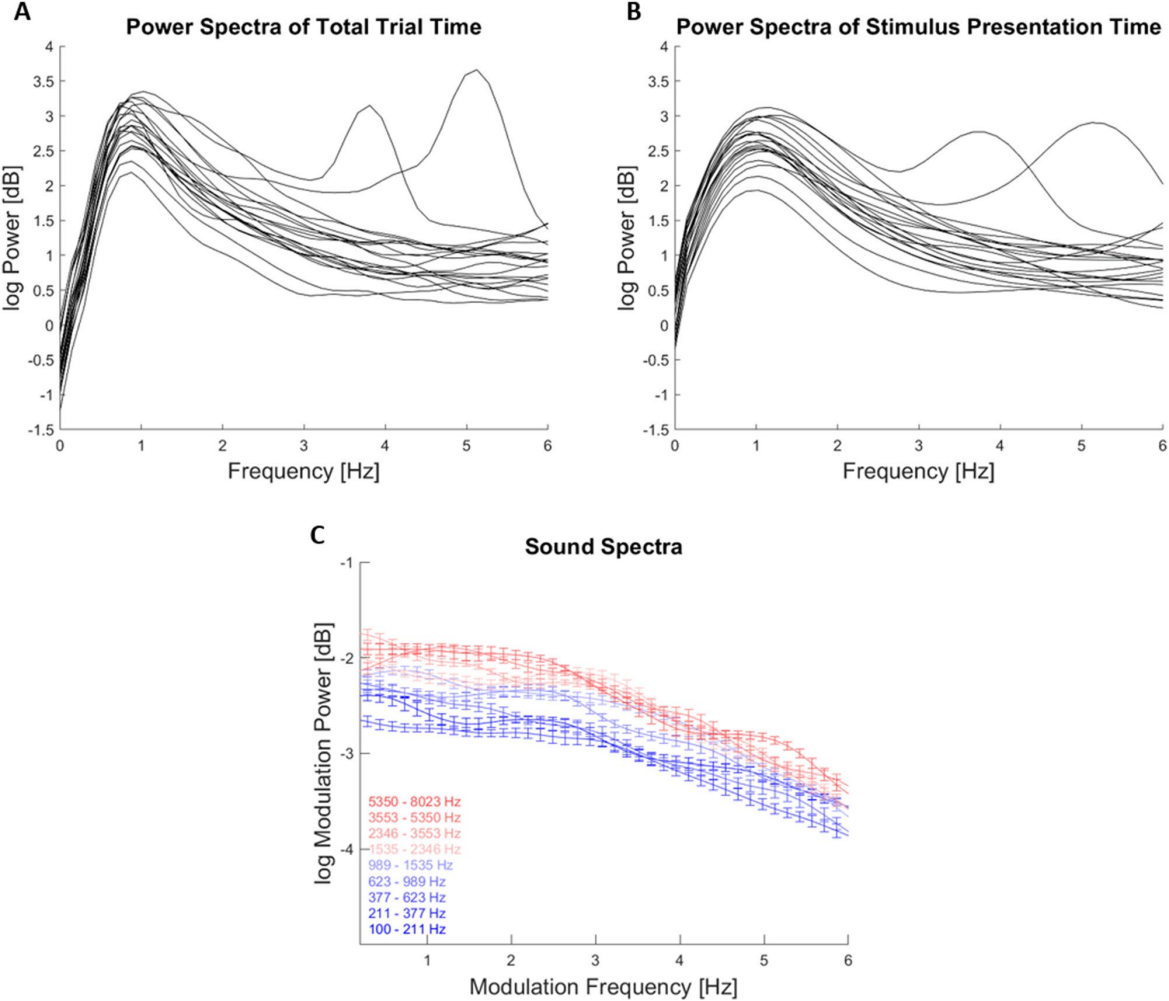
Power spectra of EEG signals and acoustic envelopes. (**A**,** B**) Time-and trial-averaged power spectra of the EEG signals, averaged within individual participants over the central electrodes contained in the significant clusters in Fig. 3 J/K. Individual lines indicate data for individual participants, in (**A**) for the entire pre-stimulus and stimulus period, in (**B**) just for the stimulus periods. (**C**) Temporal modulation spectra of the acoustic stimuli, shown for individual carrier bands (color coded). Spectra were averaged across trials for each participant. Lines and error bars indicate the mean and s.e.m. across participants.

As control analyses we investigated the frequency spectra of the EEG signals and of the envelopes of the soundscapes (Fig. 6). The time- and trial averaged EEG spectra for most individual participants were devoid of obvious peaks in the relevant frequencies, both when computed for the entire pre-stimulus and stimulus periods and when computed just for the stimulus presentation time (Fig. 6A,B). This suggests that the observed perceptual sampling around 2 Hz and the link of this to EEG activity around 4 Hz is not tied to obvious rhythmic neurophysiological signals at these frequencies. Given that the temporal structure of acoustic envelopes imprints on auditory cortical activity, we also investigated the temporal modulation spectra of the stimuli used in this experiment (as already done previously^29^). These modulation spectra (Fig. 6C) were similarly devoid of obvious spectral peaks, suggesting that the rhythmic perceptual sampling is not directly driven by a regular structure in the stimulus at the same timescales.

## Discussion

We investigated whether brain activity prior to a stimulus influences the manner in which human participants use the moment by moment acoustic evidence to make a perceptual judgement pertaining to temporally extended auditory scenes. Confirming previous results, we found that participants sample acoustic evidence not uniformly^29^; rather, the weights characterizing the perceptual sampling of random-tone acoustic soundscapes revealed a rhythmic pattern at a frequency of about 2 Hz. Importantly, the phase of this perceptual sampling co-varied with the state of pre-stimulus brain activity around 4 Hz, suggesting that the rhythmic sampling process observed in the behavioral data is directly linked to ongoing (rhythmic) brain activity. These results support the notion that the state of delta/theta band brain activity shapes the manner by which subsequent acoustic evidence influences auditory perception. In addition, and independent of the time course of perceptual sampling, we also found that the overall perceptual performance varied with the strength of pre-stimulus alpha band power over frontal electrodes, demonstrating a general influence of pre-stimulus activity on the perception of prolonged sounds.

### Evidence for the rhythmic sampling of auditory scenes

The present study capitalized on a previously developed paradigm and the present data reproduce our previous results^29^. In particular, we show that the perceptual weights attributed to different epochs in temporally extended soundscapes (> 1 s duration) composed of multiple simultaneous sequences of random tones exhibit a rich temporal structure. This structure included but was not limited to a linear trend and a significant rhythmic component at a group-level frequency of 2.2 Hz. Linear trends, in particular a decreasing influence of sensory evidence on participants choice is often seen in decision making tasks^60^. The presence of a rhythmic component is supported by two different approaches to determining whether including any rhythmic structure better explains the perceptual weights than models not containing such rhythmic structure. For the same soundscape duration (Experiment 3 in Kayser et al.^29^) the previous study revealed a very similar sampling frequency of 2 Hz. Importantly, the acoustic soundscapes used in this experiment are devoid of specific rhythmic structure at this time scale, as shown by the analysis of the frequency and temporal modulation spectra of these soundscape (Fig. 6). This suggests that the apparent rhythmicity in perceptual sampling is driven by endogenous rather than directly stimulus driven mechanisms operating at the delta band time scale.

However, the analysis of the behavioral data in isolation necessitates the assumption that the perceptual weight attributed to each epoch is fixed across trials. This is because the perceptual weight is estimated by combining the sensory information and behavioral outcome across trials. However, this assumption may not be valid, in particular if the hypothesized link between delta/theta band brain activity and rhythmic modes of perception is correct. To overcome this limitation, we here combined single trial estimates of pre-stimulus brain activity with the psychophysical reverse correlation estimate.

### Pre-stimulus activity shapes the timing of perceptual sampling

Our results directly reveal a correlation between the power and phase of pre-stimulus delta/theta band activity around 4 Hz and the relative timing of the subsequent perceptual weighting profile. Thereby our results provide a direct link between the pre-stimulus brain state and the subsequent exploitation of acoustic information for active listening over prolonged epochs (> 1 s). At the same time, we note that the strength of the phase-shift in perceptual sampling across opposing power or phase bins of the EEG activity was variable across participants (ranging from 5° to 138° for power, and from 39° to 180° for phase). This suggests that the underlying effect is either highly variable across participants or that the effect sizes obtained in the present study are still limited by number of trials collected.

Previous work has linked auditory delta band activity with changes in both spontaneous and stimulus driven neural activity^21,^^30,^^61^. The engagement of delta band activity has been implied in the attentional filtering of soundscapes and the task-relevant chunking of speech sounds into sentence or word-level structures^62–64^, and plays a central role in theories of rhythmic modes of listening^2,^^17,^^20^. However, a critical hypothesis emerging from these studies had not been tested: that rhythmic pre-stimulus activity is directly linked to the subsequent perceptual use of acoustic information over prolonged time scales. While some studies have shown the persistent fluctuations of behavioral performance at similar time scales subsequent to a brief stimulus, no study has shown that pre-stimulus activity shapes how temporally extended soundscapes are sampled to make a perceptual decision. Rather, most studies linking pre-stimulus state and qualities of perception were restricted to short (mostly 100–300 ms) stimuli^8,^^12,^^14,^^65,^^66^. We here close this gap by directly linking pre-stimulus activity and the subsequent perceptual influence of this.

We can only speculate as to why the precise time scales differed at which pre-stimulus brain state (4–5 Hz) and the perceptual sampling (2.2 Hz) were related. Spectral peaks in EEG derived brain signals are effectively blurred, both by the superposition of multiple neurophysiological sources giving rise to a particular extracranial signal and by methodological constraints in the spectral estimation processes^67^. Similarly, the spectral resolution of the perceptual weights is effectively limited by the number of trials used to estimate these and their specific parameters, such as the duration of the epochs used to randomize the sensory evidence within a trial. One possibility is hence that the underlying processes effectively operate at very much the same time scales, which simply emerge differently given experimental constraints. Alternatively, it could be that the relevant neurophysiological pre-stimulus processes and the perceptual sampling are directly linked and share a common time scale, but this time scale is slowed down during stimulus presentation, and hence appears distinct in the present analysis. Future studies are required to investigate these questions in more detail.

A number of studies have linked delta and theta band activity to processes mediating the prediction of upcoming stimuli^68,69^. Given that the stimuli in the present paradigm were presented following a fixation cue, it is possible that the pre-stimulus EEG signatures related to the perceptual sampling of the acoustic evidence are shared with those implied in predictive processes. In fact, such a link would not be surprising, as delta/theta band activity has also been implied in mediating predictions in speech sounds based on acoustic, prosodic or phonetic features^15,70^, and hence the sampling of acoustic scenes may be tightly related to the search or exploration of temporally predictive structures. We did not observe systematic peaks in the EEG spectra in the delta/theta band across participants, which would be indicative of an obvious process specifically represented at these frequencies. However, few studies so far have directly linked the observed relation of delta/theta band phase to specific spectral peaks in M/EEG signals, a future work is required to understand the precise neurophysiological processes giving rise to the perceptual sampling investigated here.

### Pre-stimulus power shapes behavior

We also found a significant relation between pre-stimulus alpha (8–13 Hz) power and participants' sensitivity to the direction of acoustic sweep. Generally, such a relation of pre-stimulus power and perceptual performance has been observed in a wide range of perceptual studies across sensory modalities^8,14,71–76^. However, most studies in the auditory domain reported effects predominantly at lower delta or theta band frequencies^8,9,11–14,77^, while a role for alpha band activity is typically discussed for vision and spatial attention paradigms. Though, some studies have linked alpha power to auditory perception^78–80^.

Previous work on the entrainment of brain activity to speech has shown that frontal alpha band power correlates with the strength of delta band speech-tracking^21^. Frontal alpha could reflect a mechanism that shapes the alignment of rhythmic activity in auditory regions to the acoustic stimulus in a top-down manner^81,82^. Given that stronger speech-to-brain alignment is also predictive of improved speech reception^83,84^ these results suggest that frontal alpha power may be generally predictive of the correct identification of complex sounds. The positive relation of pre-stimulus alpha and improved sensitivity observed here further supports for this notion, although we did not find a significant relation between alpha power and the perceptual weights themselves.

Alternatively, these discrepancies in perceptually-relevant EEG frequencies in the present and previous work could be explained by the rather long duration of the soundscapes used here. While the soundscapes used here lasted more than a second, previous studies reporting a correlation of pre-stimulus power and behavioral outcome mostly used brief stimuli (e.g. < 200 ms)^8,14^. Hence, one cannot rule out that the previously observed effects in delta/theta bands and the alpha effect shown here reflect two distinct neurophysiological mechanisms that each shape perception for shorter and longer stimuli, respectively.

## Conclusion

We systematically investigated the relation between pre-stimulus brain activity and rhythmic perceptual sampling of long and non-rhythmic stimuli. Our data show that strength and the timing of delta/theta band pre-stimulus EEG activity relates to the rhythmic perceptual sampling of auditory scenes. These results directly point to a lasting influence of spontaneous rhythmic brain activity for the perception of subsequent stimuli and close a critical gap in the conceptual picture proposing a fundamental role of rhythmic auditory cortical activity for active listening.

## Data Availability

The original and preprocessed data and Matlab code are available upon request.
